# Analysis of sterilizer allocation and related factors in dental health-care settings in Yunnan Province, China: a cross-sectional study

**DOI:** 10.1186/s12903-024-04215-8

**Published:** 2024-04-05

**Authors:** Xinchun Zou, Haiyan Ding, Qi Sun, Wen Lin Lu, Ying Liu, Shinan Zhang, Zhangcheng Yin, Congchong Shi, Guozhong He, Ching-Wen Chien, Jie Liu, Juan Liu

**Affiliations:** 1 Department of Infection Management, Kunming Medical University School and Hospital of Stomatology, Kunming, 650106 China; 2 Cancer Biotherapy Center, Key Laboratory of Melanoma Research, Peking University Cancer Hospital Yunnan (Yunnan Cancer Hospital,The Third Affiliated Hospital of Kunming Medical University), Kunming, 650031 China; 3 Department of Preventive Dentistry, Kunming Medical University School and Hospital of Stomatology, Kunming, 650106 China; 4 Department of the Second Clinic, Kunming Medical University School and Hospital of Stomatology, Kunming, 650106 China; 5 Department of General Office, Kunming Medical University School and Hospital of Stomatology, Kunming, 650106 China; 6 Department of Orthodontics, Kunming Medical University School and Hospital of Stomatology, Kunming, 650106 China; 7 School of Public Health, Kunming Medical University, Kunming, China; 8 Institute for Hospital Management, Tsing Hua University, Shenzhen Campus, 518055 China; 9 People’s Liberation Army of China General Hospital, Beijing, 100039 China

**Keywords:** Disinfection and sterilization, Dental health-care settings, Allocation of equipment, Dental unit

## Abstract

**Objective:**

To investigate the status and related factors of sterilizers in dental health-care settings in Yunnan Province, with the aim of providing a theoretical basis for the health administrative department to formulate regional quality control programs and systems, proposing reasonable suggestions for optimizing the allocation of sterilizer resources in Yunnan’s dental health-care settings, thereby improving resource utilization efficiency.

**Methods:**

This cross-sectional survey was conducted in 2600 dental health-care settings in Yunnan Province in March 2020. Uni-variable linear regression, multi-variable linear regression, curve fitting and threshold effect analysis were used to understand the relationship between dental units and sterilizers.

**Results:**

A total of 2600 dental health-care settings were included. The disinfection and sterilization work were mainly completed by the dental department in 1510(58.1%) institutions. 44(1.7%) institutions were not allocated sterilization equipment, and 1632 (62.8%) had only one sterilizer. The median allocation of sterilizers was 1.0. Uni-variable linear regression showed significant differences in covariates such as dental unit, dental handpiece, disinfection equipment, dentist, and dental assistant, which were more sensitive (*p* < 0.001) and statistically significant. The adjusted model was more stable in the multi-variable linear regression, and the differences in covariates between different settings were statistically significant. Curve fitting revealed an S-shaped curvilinear relationship between the number of dental units and sterilizers in oral healthcare settings.

**Conclusion:**

The disinfection and sterilization work was mainly completed by the dental department in dental health-care settings in Yunnan Province. Sterilizer allocation increases with the number of dental units, but some institutions have insufficient allocation of sterilizer and manpower resources, resulting in certain risks of infection control. Thus, it is necessary to strengthen supervision, inspection and regional quality control work in infection control of dentistry.

**Supplementary Information:**

The online version contains supplementary material available at 10.1186/s12903-024-04215-8.

## Introduction

Under the influence of the COVID-19 pandemic, government departments and medical institutions have paid more attention to infection control [1], and public awareness of infection control has also been awakened [2]. More people are focusing on infection control [3], which has increased the pressure and challenges in this field [4]. Dental healthcare providers (DHCP) may contact pathogens while administering dental treatment [5], either through handling contaminated equipment or being exposed to blood and respiratory secretions [6]. Due to its specificity, infection control work in the dental practice is more important and valued by patients than ever before [7]. Instrument disinfection and sterilization are the core link of infection control, and to a large extent, reflect the infection control level of an medical institution. The sterilizer is the responsible instrument for the sterilization of dental instruments [8], which is one of the most critical equipment for the opening of dental health-care settings, as well as one of the most important indicators for evaluating the quality of infection control work in dental institutions. However, some dental health-care settings have insufficient sterilizer allocation and cannot meet the needs of diagnosis and treatment, or they have no cooperation with the third-party central sterile supply department (CSSD),all of which exists potential safety hazards.

### Objectives

To grasp the allocation of sterilizers in dental health-care settings and to understand the influencing factors of sterilizer allocation in Yunnan Province, in early 2020, the Yunnan Oral Disease Diagnosis and Treatment Quality Control Center conducted the “Survey of Dental Medical service capabilities of Yunnan Province”, which included the situation of disinfection and sterilization in dental health-care settings in Yunnan Province.

This project indicated that that the allocation of sterilizers is the key to infection control in dental institutions, and whether the allocation is adequate needs to be comprehensively considered with other factors. Therefore, allocation ideas for enhancing the level and capacity of regional infection control can be proposed by analyzing the allocation of sterilizers in dental health-care settings in Yunnan Province and its influencing factors. This article can also provide decision-making references for dental institutions and relevant managers in other areas.

## Materials and methods

### Study design

This was a cross-sectional study that was conducted to investigate all dental medical institutions to understand the allocation of sterilizers in Yunnan Province.

### Setting

Setting: In 2020, the Yunnan Oral Disease Diagnosis and Treatment Quality Control Center, in collaboration with the Yunnan Provincial Stomatological Association, led the “Survey of Dental Medical service capabilities of Yunnan Province” project on behalf of the Yunnan Provincial Health Commission. The deadline of this survey was December 31, 2020, and included all medical institutions that provide dental health care services within Yunnan Province and were registered with the health administrative department (“ dental health-care settings”).

### Participants

This cross-sectional study targeted all institutions providing dental health care services in Yunnan Province. As long as an institution provided dental services, the principle of the institution should reported data, and local health administrative departments should supervise the reporting. The project team verified the data through on-site inspections and follow-up calls.

### Variables

This study focused on the allocation and influencing factors of sterilizers in dental health-care settings. Relevant factors included dental handpieces, dentists, dental assistants (nurses with specialized dental training), and disinfection equipment.

This study focuses on sterilizers, the sterilizers of each institution in this study is the sum of various types of sterilization equipment, including large steam sterilizer, small steam sterilizer, ethylene oxide sterilization, and low-temperature plasma sterilizer. Large steam sterilizer volume greater than 60 l, small steam sterilizer less than 60 l. Small steam sterilizers include type B and type N sterilization cycles. The disinfection equipment includes mechanical cleaning and disinfection equipment and ultrasonic cleaners. Disinfection is done by damp heat and ultrasonic.

The other variables were state, nature of institutions, type of institution, and sterilization providing.

The quantitative variables in this study included dental unit, handpiece, sterilization, disinfection, dentists, dental assistants, etc. Dental institutions were categorized into four sizes according to the number of dental units (≤2, 3-4, 5-20, ≥20).

### Data sources

All data in this study were directly reported by dental health-care settings through online questionnaires, and the data were registered by the settings themselves.

### Bias

This study might have non-response bias and reporting bias, and corresponding measures were taken to exclude them. (1) To reduce non-response bias, the project was uniformly issued by Yunnan Provincial Health Commission, requiring all local dental health-care settings to fill in on time, and the project team checked the institution directory and verified the filling situation. The institutions that did not fill in were urged by phone, but there were a small number of institutions that did not fill in data as needed. (2) To reduce reporting bias, a few institutions did not fill in the questionnaire seriously, and project team members checked and verified all data one by one. Abnormally reported data were verified again by phone, and some areas used on-site inspections to eliminate reporting bias.

### Study size

This study planed to collect full-sample dental health-care settings data from the entire province, and after multiple efforts, a total of 2712 institutions have reported data, which were basically in line with the institution directory provided by the Health Commission. It covers data from dental health-care settings in 16 prefectures in the province and finally confirms 2600 valid data points according to the inclusion criteria.

### Statistical analysis

All of the analyses were carried out using the statistical software programs R (2The R Foundation) and Free Statistics software version 1.8 (Yang et al., 2021). Mean ± standard deviation and frequencies (percentages) were used to describe demographic and clinical data. The T-test was used to analyze the normal distribution, and the Kruskal–Wallis test was used to analyze the skewed distribution in continuous variables. Uni-variable linear regression, multi-variable linear regression, curve fitting, threshold effect analysis and other methods were used to understand the relationship between dental units and sterilizers. The β-value and corresponding 95% CI were estimated for the allocation and related factors of sterilizers in oral institutions. *P* values < 0.05 were considered statistically significant.

## Results

### Data collection and exclusion

**Fig. 1:**
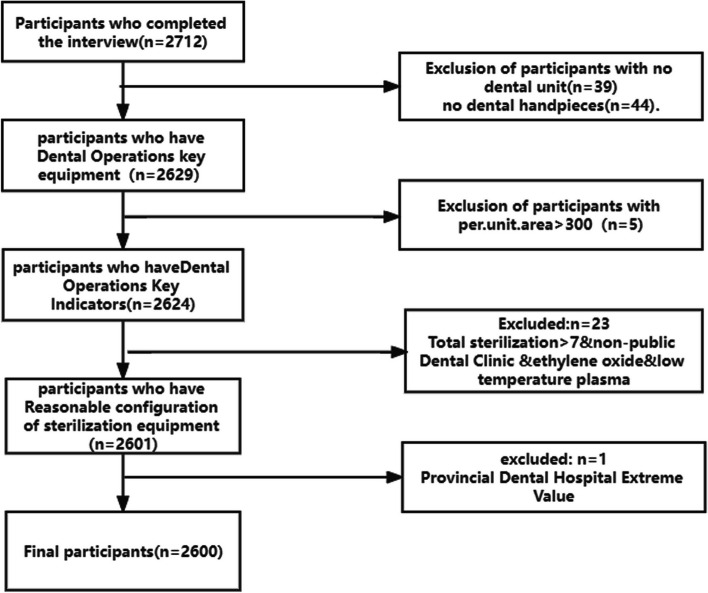
Flow chart of the study

During the project execution period, 2712 dental settings were collected by project teams, and after repeated checks, some abnormal data were revised by phone verification. Abnormal data that could not be verified and revised were excluded according to the following criteria: 1. The reporting institution had no key equipment, such as dental units or dental handpieces. 2. There were data that did not conform to logic or common sense. For example, after comparing the reported data, some institutions had each dental unit occupying an area of more than 300 square meters, and some nonpublic medical institutions had more than 7 sterilizers and were equipped with ethylene oxide and low-temperature plasma sterilizers. 3. The Affiliated Stomatology Hospital of Kunming Medical University (KMU) is a provincial specialized stomatological hospital with different sizes and natures, so it was excluded as an extreme value. A total of 2600 dental institutions’ data were included in this study (Fig. 1).

### Baseline characteristics of participants

The distribution of dental health-care settings in Yunnan Province varies greatly, Kunming owned the most, with 1106 (42%); Nujiang and Diqing states owned the fewest, with only 19(0.7%) each(*p* < 0.001).

From institution type, most of the institutions 1406 (54.1%) were dental clinics with, relatively few were oral specialized hospitals, only 23 (0.9%) (*p* < 0.001).

From disinfection and sterilization services, 1510 (58.1%) institutions carried out disinfection and sterilization work in dentistry, 731 institutions (28.1%) underwent disinfection in the central sterile supply department (CSSD), and 359 institutions (13.8%) jointly undertook disinfection work with the dental department and CSSD (*p* < 0.001).

From equipment allocation, dental units, handpieces, sterilizers, and disinfection equipment allocations presented skewed distributions, and the allocation of different sizes of dental institutions had statistically significant difference (*p* < 0.001); the larger the size of the allocation was.

From dental healthcare personnel (DHCP): Dentist and dental assistant staff showed skewed distribution, with a statistically significant difference in DHCP between different sizes of dental institutions, *p* < 0.001, with more staffs at larger sizes of dental institutions (Table 1).

**Table 1:** Baseline characteristics of participants

Variables	Total (*n* = 2600)	size1 (*n* = 601)	size2 (*n* = 1290)	size3 (*n* = 682)	size4 (*n* = 27)	*p*-value
State, n (%)						< 0.001
Kunming	1106 (42.5)	373 (62.1)	492 (38.1)	229 (33.6)	12 (44.4)	
Qujing	236 (9.1)	41 (6.8)	122 (9.5)	71 (10.4)	2 (7.4)	
Honghezhou	184 (7.1)	26 (4.3)	92 (7.1)	62 (9.1)	4 (14.8)	
Dali	173 (6.7)	29 (4.8)	80 (6.2)	63 (9.2)	1 (3.7)	
Yuxi	165 (6.3)	15 (2.5)	92 (7.1)	57 (8.4)	1 (3.7)	
Wenshanzhou	114 (4.4)	24 (4)	61 (4.7)	29 (4.3)	0 (0)	
Chuxiongzhou	110 (4.2)	10 (1.7)	62 (4.8)	38 (5.6)	0 (0)	
Zhaotong	88 (3.4)	20 (3.3)	49 (3.8)	19 (2.8)	0 (0)	
Lincang	79 (3.0)	15 (2.5)	41 (3.2)	20 (2.9)	3 (11.1)	
Lijiang	73 (2.8)	8 (1.3)	52 (4)	13 (1.9)	0 (0)	
Xishuangbanna	66 (2.5)	6 (1)	46 (3.6)	14 (2.1)	0 (0)	
Baoshan	58 (2.2)	8 (1.3)	25 (1.9)	24 (3.5)	1 (3.7)	
Dehong	56 (2.2)	8 (1.3)	30 (2.3)	17 (2.5)	1 (3.7)	
Pu′er	54 (2.1)	12 (2)	20 (1.6)	20 (2.9)	2 (7.4)	
Nujiang	19 (0.7)	2 (0.3)	15 (1.2)	2 (0.3)	0 (0)	
Diqing	19 (0.7)	4 (0.7)	11 (0.9)	4 (0.6)	0 (0)	
**Nature of institutions, n (%)**						< 0.001
Public	596 (22.9)	195 (32.4)	215 (16.7)	172 (25.2)	14 (51.9)	
Nonpublic	2004 (77.1)	406 (67.6)	1075 (83.3)	510 (74.8)	13 (48.1)	
**Type of institution, n (%)**						< 0.001
General Hospital Stomatology	492 (18.9)	67 (11.1)	230 (17.8)	182 (26.7)	13 (48.1)	
General Clinic Stomatology	271 (10.4)	175 (29.1)	89 (6.9)	7 (1)	0 (0)	
Primary Care Stomatology	402 (15.5)	230 (38.3)	155 (12)	17 (2.5)	0 (0)	
Stomatological Hospital	23 (0.9)	0 (0)	1 (0.1)	11 (1.6)	11 (40.7)	
Dental Clinic	1406 (54.1)	126 (21)	813 (63)	464 (68)	3 (11.1)	
Others	6 (0.2)	3 (0.5)	2 (0.2)	1 (0.1)	0 (0)	
**Provide sterilization, n (%)**						< 0.001
Dentistry	1510 (58.1)	324 (53.9)	854 (66.2)	324 (47.5)	8 (29.6)	
Central Sterile Supply Department	731 (28.1)	201 (33.4)	294 (22.8)	226 (33.1)	10 (37)	
Comanagement	359 (13.8)	76 (12.6)	142 (11)	132 (19.4)	9 (33.3)	
**Sterilizers, n (%)**						< 0.001
0	44 (1.7)	21 (3.5)	18 (1.4)	4 (0.6)	1 (3.7)	
1	1632 (62.8)	492 (81.9)	858 (66.5)	281 (41.2)	1 (3.7)	
2	518 (19.9)	54 (9)	227 (17.6)	233 (34.2)	4 (14.8)	
3	217 (8.3)	34 (5.7)	92 (7.1)	84 (12.3)	7 (25.9)	
4	62 (2.4)	0 (0)	30 (2.3)	27 (4)	5 (18.5)	
5	99 (3.8)	0 (0)	56 (4.3)	40 (5.9)	3 (11.1)	
>5	28 (1.1)	0 (0)	9 (0.7)	13 (1.9)	6 (22.2)	
Dental unit, Median (IQR)	2.0 (2.0, 4.0)	1.0 (1.0, 1.0)	2.0 (2.0, 3.0)	5.0 (4.0, 7.0)	24.0 (20.0, 35.5)	< 0.001
Total handpieces, Median (IQR)	23.0 (11.0, 40.0)	8.0 (4.0, 13.0)	21.0 (13.0, 31.0)	47.0 (32.2, 71.8)	139.0 (92.5, 270.5)	< 0.001
Disinfection, Median (IQR)	1.0 (0.0, 3.0)	1.0 (0.0, 2.0)	1.0 (0.0, 3.0)	2.0 (1.0, 3.0)	4.0 (2.0, 5.0)	< 0.001
Total sterilizers, Median (IQR)	1.0 (1.0, 2.0)	1.0 (1.0, 1.0)	1.0 (1.0, 2.0)	2.0 (1.0, 2.0)	4.0 (3.0, 5.0)	< 0.001
Dentists, Median (IQR)	2.0 (1.0, 3.0)	1.0 (1.0, 1.0)	2.0 (1.0, 2.0)	4.0 (3.0, 6.0)	16.0 (10.5, 24.5)	< 0.001
Dental Assistant, Median (IQR)	1.0 (1.0, 3.0)	1.0 (0.0, 1.0)	1.0 (1.0, 2.0)	4.0 (2.0, 6.0)	18.0 (12.5, 21.5)	< 0.001

*P*-values were calculated using chi-square test and Kruskal-Wallis test

### Outcome data

The distribution of dental units presented a skewed pattern, with a median of 2 and inter-quartile ranges of 2 and 4. Based on this, the size of dental institutions could be divided as follows: Size 1 (≤2 dental units), Size 2 (3-4 dental units), Size 3 (5-20 dental units), and Size 4 (≥20 dental units). If a dental setting has 20 or more dental units, it should be managed as a secondary specialized hospital.

**Fig. 2:**
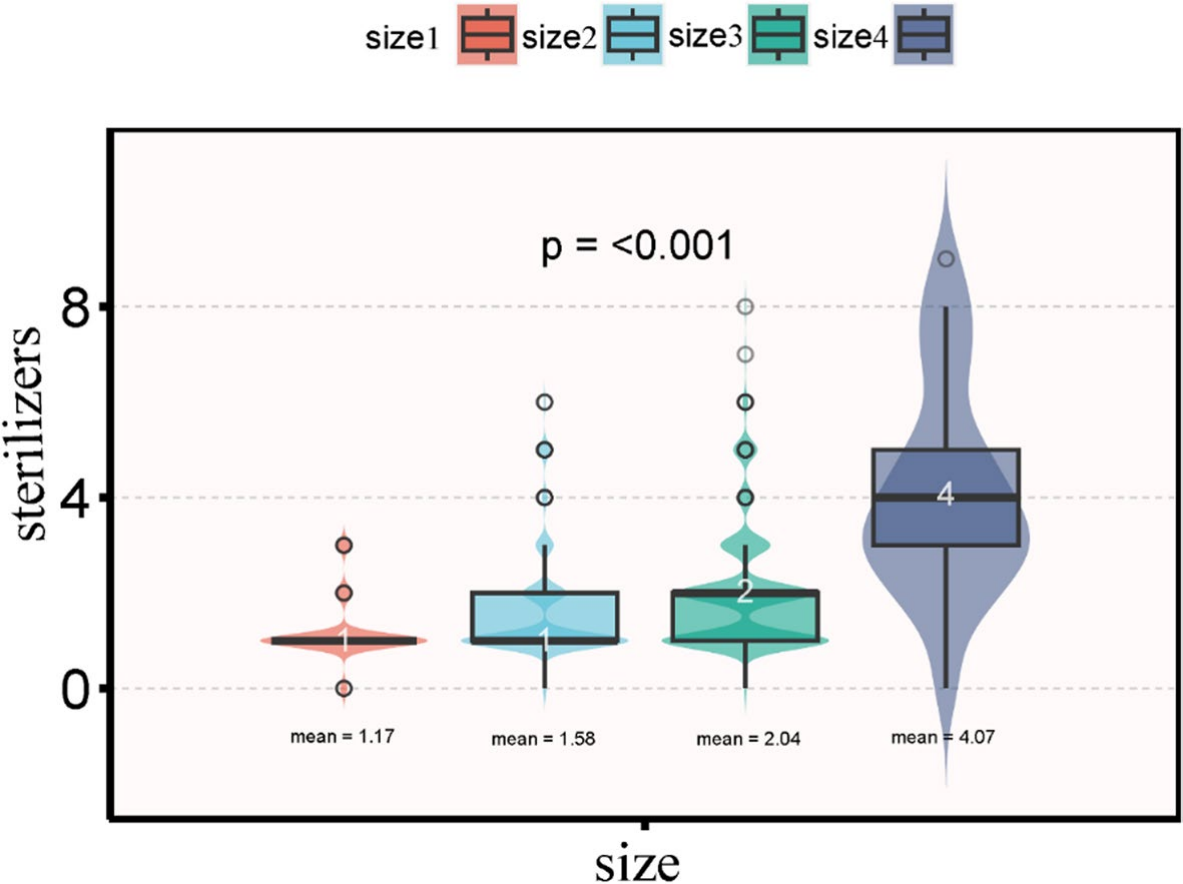
Different sizes of dental health-care setting sterilizer allocation

Table 1 and Fig. 2 show significant difference in the allocation of sterilizer devices among institutions of different sizes. The distribution of d sterilizers follows a skewed pattern, with a median of 1 and inter-quartile ranges of 1 and 2. The mean value of sterilizer allocation was 1.17 for size 1 dental settings in Yunnan Province, 1.58 for size 2, 2.04 for size 3, and 4.07 for size 4 (*p* < 0.001) (Table 1, Fig. 2).

The results showed that the majority of dental settings were small-scale, with a total of 1891 settings having 4 dental units or less, and only a few settings had 20 or more dental units. In regard to providing disinfection and sterilization services, most settings choose to perform them within dental clinics, totaling 1510 (58.1%), with small-scale institutions being the majority. In terms of sterilizer allocation, 44 settings do not have any sterilizers, with small-scale institutions being the majority. Most settings (1632) only have one sterilizer. As the scale of the setting increases, the number of sterilizers also increases.

### Main results

#### Sensitivity analysis and subgroup analysis

There were significant differences between the dental healthcare facilities in terms of dental units, handpieces, sterilizers, disinfection equipment, dentists, and dental assistants, and the sensitivity of the indicators was higher, *p* < 0.001, all of which were statistically significant (Table 2).

**Table 2:** Uni-variable linear regression models evaluating the association between basic variables and sterilizers

Item	β.(95%CI)	*p*-value
Dental unit	0.1 (0.09,0.11)	< 0.001
Total handpieces	0.01 (0.01,0.01)	< 0.001
Disinfection	0.31 (0.28,0.33)	< 0.001
Dentists	0.12 (0.11,0.13)	< 0.001
Dental Assistant	0.09 (0.08,0.1)	< 0.001

In the multi-variable linear regression, the allocation of sterilizers was mainly related to the size of the institution, dental units largely responded to the size of the institution, and at the same time, the allocation of the sterilizers was also affected by other related factors. Therefore, sterilizers and dental units were subjected to a multi-variable linear regression analysis, and the β value was 1.3 before adjustment. The 95% confidence interval was 1.25 ~ 1.36, and the model was adjusted to gradually increase the covariates. The model was more stable, with *p* < 0.001, which was statistically significant (Table 3).

**Table 3:** Multi-variable linear regression models evaluating the association between dental unit and sterilization

Variable	β_95CI	*p*-value
Non adjusted Model	1.3 (1.25 ~ 1.36)	< 0.001
Model I	1.26 (1.21 ~ 1.32)	< 0.001
Model II	0.89 (0.83 ~ 0.95)	< 0.001
Model III	0.89 (0.83 ~ 0.95)	< 0.001

Model I, Adjusted for total.handpieces

Model II, Adjusted for Model I and disinfection

Model III, Adjusted for Model II, and Dentists, Dental. Assistant

#### Threshold effect analysis

**Fig. 3:**
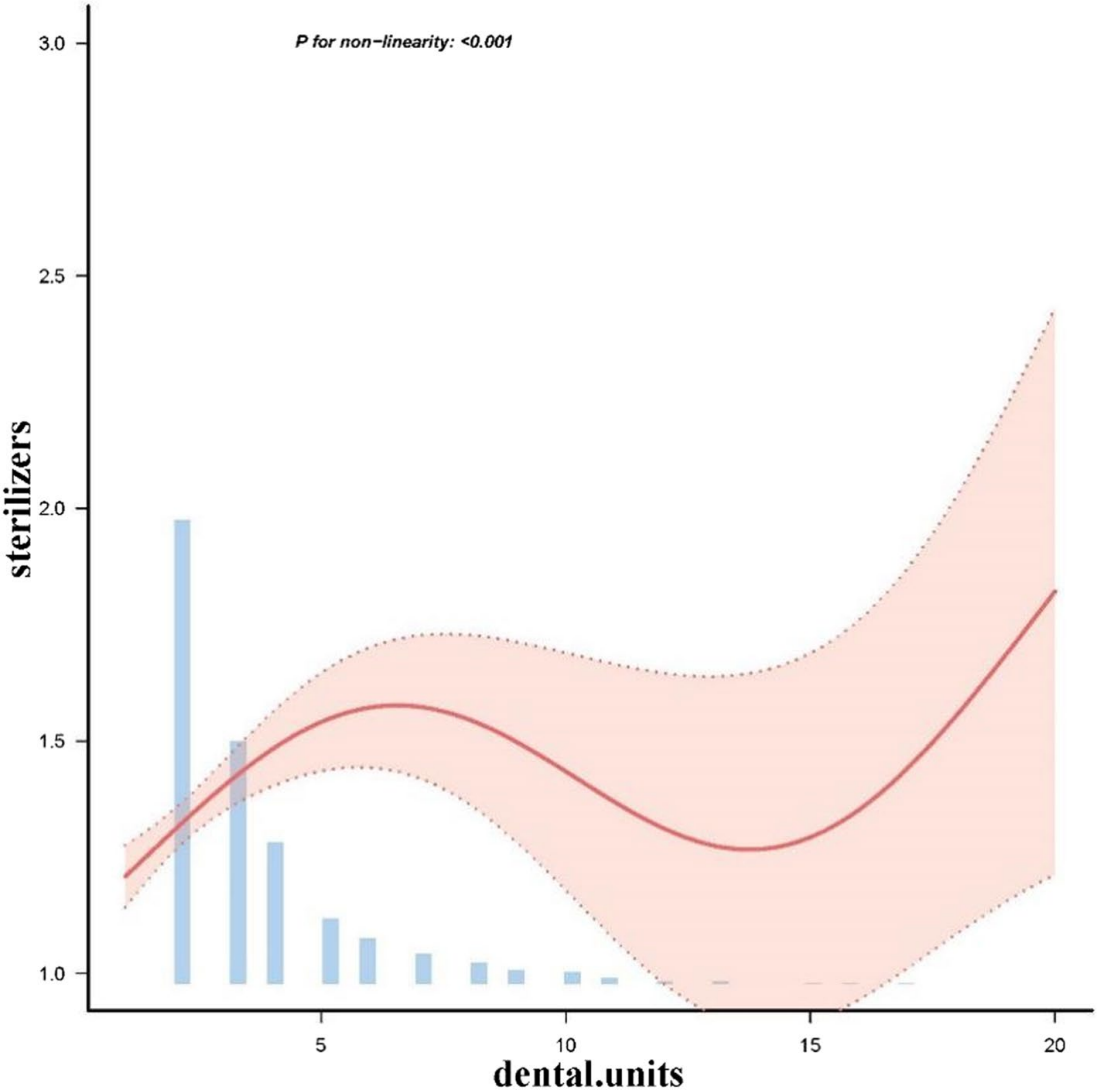
Smooth curve fitting of the relationship between sterilizers and dental units. Only 99% of the data are displayed. Adjustment factors included handpieces, disinfection, dentists, and dental assistants

Curve fitting indicates that the allocation of sterilizers and dental units was nonlinear (*p* value, nonlinear<0.001) but curved, and a model using restricted cubic splines with four knots revealed an S-shaped association, with an increasing sterilizer as the dental units increased from 0 to 6.0 (β0.08 [0.036-0.123]). When dental units were beyond 6, the sterilizers increased which not showed significantly change (Fig. 3, Table 4). In the figure, the pink solid line indicated the estimated allocation of sterilizers, and the light pink shadow represented the point wise 95% CI adjusted for included handpieces, disinfection, dentists, and dental assistants. In small-scale dental institutions with fewer dental units, there was a clear concentration trend, most of them equipped with one small sterilizer. As the size increased, the sizes and models of the purchased sterilizers vary, resulting in different quantities. Overall, the allocation quantity shows a growing trend (Fig. 3).

**Table 4:** Threshold analyses of sterilizers and dental units using two piece-wise regression models

Threshold of sterilizers	β	95% CI	*p*-value
Dental units<6	0.08	0.036-0.123	< 0.001
Dental units<15	− 0.05	−0.164-0.065	0.395
Dental units≥15	0.184	−0.132-0.501	0.241
Likelihood Ratio test	–	–	0.014

*CI* Confidence interval. Adjustment factors included handpieces, disinfection, dentists, and dental assistants

## Discussion

The majority of dental healthcare institutions in Yunnan Province are small-scale, with a main investment in essential equipment such as dental units and handpieces. However, the allocation of sterilizers were relatively inadequate, which presents potential threats to the disinfection and sterilization of instruments. Chemical disinfection and sterilization methods were selected for dental healthcare institutions without sterilizers. Transmission may result such as the use of improperly sterilized medical or dental equipment [9]. Based on fig. 2 (violin plots), it is determined that institutions of size 1 and size 2 should have at least one sterilizer, but size 2 is recommended to allocate 2 sterilizers, size 3 should have at least 2 sterilizers, and size 4 should have at least 4 sterilizers. For larger dental institutions, massive steam sterilizers were advised. Among the 2600 dental institutions, at least 337 (12.9%) are found to have insufficient allocation of sterilizers [10].There were infection control risks in terms of the allocation of sterilizers.

It is recommended to standardize the allocation of sterilizers and to use high-temperature and high-pressure steam sterilization methods for dental instruments [11]. Institutions should configure sterilizers depending on their business volume. Newly established oral institutions should require preapproval for the allocation of cleaning and sterilizers based on their needs. As long as oral medicine are provided and dental instruments are reused, there should be at least one sterilizer, and the configuration of sterilizers should be increased as the number of dental units increases. Through curve fitting analysis, it was observed that as the scale of the institution expands and the number of dental units increases, the quantity of sterilizer follows an S-shaped curve. It mainly increased up to 6 dental units and then decreased as the number of treatment units continues to increase. After verification, it was found that institutions, after expanding their scale, begin to purchase larger capacity sterilizers. As the sterilization requirements are met, the quantity of equipment decreases. Larger institutions generate more daily sterilization items, requiring more equipment to meet the demand [12].

The sterilizer allocation should also take into account parameters such as the number of dental instruments and DHCP, and be designed with an appropriate sterilizer and sufficient time for instrument sterilization. Allocation sterilization processes guarantee the sterility of dental instruments but can negatively affect instrument features by altering their physical and mechanical properties [13]. Paying attention to recording the use and sterilization times, if conditions can be equipped with low temperature plasma equipment, low temperature plasma technology is more suitable for dentistry [14].

Therefore, encouraging third-party CSSD to provide sterilization services could be a good way to reduce the infection control risks. For many small dental health-care settings, the cost pressure is relatively high to invest in a complete set of disinfection and sterilization equipment. The CSSD ensures the quality of medical care and controls infection [15]. Small oral institutions can obtain cost-effective benefits by providing standardized disinfection and sterilization services through third-party CSSD, recommending the centralization of reprocessing [16], providing safe reusable instruments [17], and successfully reducing the investment in disinfection and sterilization equipment. Regulatory authorities should focus on monitoring the standardized behavior of third parties while also checking cooperation agreements, supply situations between parties and verifying whether the volume of outsourced disinfection and sterilization matches the number of patients treated, to avoid fraudulent contracts and ensure that no contaminated instruments are reused. Government departments should also introduce policies to encourage eligible institutions to establish third-party CSSD to provide services to institutions in need within the region.

There are also infection control risks in terms of disinfection and sterilization work. The professional, standardized, and scientific management of the CSSD is prerequisite if a hospital is to realize sustainable development. The medical industry standard “Regulation for disinfection and sterilization technique of dental instruments” (WS 506-2016) allows dental instruments to be sterilized in dentistry or sent to the Central Sterilization Supply Department (CSSD), taking into account the special characteristics of small dental institutions. This study found differences in the department responsible for disinfection and sterilization work. In small dental healthcare settings, the cleaning, disinfection, drying, oiling, and sterilization of dental instruments were mainly carried out in the department, and the equipment for cleaning, disinfection, drying, oiling, and sterilizer is evidently insufficient, which were similar to the results in Qinghai and Ningxia. The disinfection and sterilization work of dental institutions should be further standardized, internal cleansing of handpieces were insufficient and that a final mechanical disinfection is indispensable [18], and their instrument institutions were recommend to sent to the CSSD for unified sterilization, gradually reducing the use of sterilizers in dentistry. Thus, centralized supply of sterilized instruments by CSSD is essential.

Specialized personnel should be employed for infection control work. Full-time and part-time specialized personnel were primarily responsible for sterilization and infection control. During quality control inspections, it was found that some dental practitioners had a poor sense of infection control, lack of knowledge and skills of infection control, unfamiliar with infection control requirements [19]. The administrative department and quality control department should strengthen systematic training for dental professionals, especially specialized personnel, including infection control principles, requirements for instrument disinfection and sterilization. Also including laws, regulations, industry standards and technical guidelines of infection control, to ensure the implementation of infection control measures. If the staffs cannot correctly grasp the method of disinfection and sterilization of various instruments, it may also lead to the failure of sterilization. Decontamination is the combination of processes used to make a reusable item safe for further use on patients and handling by staff [10]. Internal cleansing of handpieces is insufficient, and final mechanical disinfection is indispensable [18]. Although type B and type S autoclaves allow us to sterilize dental handpieces, it is important to realize that complete sterilization of the handpiece is not always achieved by a type N autoclave [20].

Health supervision departments should strengthen their supervision and inspection of dental healthcare settings, and the oral quality control center should play a professional role. Joint inspections by health supervision departments and quality control centers were proven to be more effective in the past, as quality control center experts were more familiar with oral infection control requirements and could find existing problems. Based on the results of quality control inspections, health supervision departments could use their enforcement powers to take action against small dental healthcare settings that do not satisfy hardware standards and cannot handle disinfection and sterilization concerns. When risks are identified, a supervisory opinion should be issued, improvement suggestions should be clearly provided to the settings, and implementation should be supervised within a limited time frame. If necessary, a follow-up inspection should be conducted to check the implementation of the supervisory opinion. Currently, China has gradually formed a five-level quality control network consisting of the National Quality Control Center, provincial quality control centers, state and city quality control centers, county-level quality control stations, and institutional quality control organizations. The quality control network should be further standardized and improved and actively fulfill its responsibilities by promoting the homogenization development of oral infection control, quality control work in corresponding regions through standard promotion, education and training, quality control inspections, and other means.

### Limitations

There were some limitations in this survey, such as information on the allocation of specialized personnel for infection control, the capabilities of these personnel, and the implementation of infection control measures were not collected. The dates in this study was reported by each oral medical institution. Relevant standards require that medical institutions should regularly monitor the functional status of sterilizer and make a registration. After the questionnaire collection, the research group randomly selected some oral institutions for on-site review to confirm the operation of sterilizer. Due to the large number of institutions, it was not possible to reviewed all institutions on site. These limitations need to be addressed and improved upon in future research.

### Supplementary Information

**Supplementary Material 1.**

## Data Availability

The datasets used and/or analysed during the current study available from the corresponding author on reasonable request.

## Abbreviations

COVID-19: Coronavirus Disease 2019
DHCP: Dental healthcare personnel
CSSD: Central sterile supply department
CI: Confidence interval
